# Supplementary material for: Mechanisms governing the pioneering and redistribution capabilities of the non-classical pioneer PU.1
Source: Nat Commun. 2020 Jan 21;11:402. doi: 10.1038/s41467-019-13960-2 (PMC6972792; doi:10.1038/s41467-019-13960-2)
Supplement: Supplementary file 7 — Source data [file 41467_2019_13960_MOESM7_ESM.zip › Source_Data/FigureS2/FigureS2B_MotifScanOutput/KmeansCluster1/homerResults/motif4.similar.html]

motif4

## Information for motif4

C
T
G
A
T
C
G
A
T
C
A
G
C
T
A
G
T
C
A
G
C
T
A
G
T
C
G
A
T
C
G
A
T
C
A
G
  
Reverse Opposite:  

A
G
T
C
A
G
C
T
A
G
C
T
G
A
T
C
A
G
T
C
G
A
T
C
A
G
T
C
A
G
C
T
G
A
C
T
  

|  |  |
| --- | --- |
| p-value: | 1e-630 |
| log p-value: | -1.453e+03 |
| Information Content per bp: | 1.553 |
| Number of Target Sequences with motif | 2478.0 |
| Percentage of Target Sequences with motif | 48.67% |
| Number of Background Sequences with motif | 5625.4 |
| Percentage of Background Sequences with motif | 14.18% |
| Average Position of motif in Targets | 104.1 +/- 49.4bp |
| Average Position of motif in Background | 101.4 +/- 88.7bp |
| Strand Bias (log2 ratio + to - strand density) | -0.1 |
| Multiplicity (# of sites on avg that occur together) | 1.90 |
| Motif File: | file (matrix) reverse opposite |

### Similar de novo motifs found

|  |  |  |  |  |  |  |  |
| --- | --- | --- | --- | --- | --- | --- | --- |
| Rank | Match Score | Redundant Motif | P-value | log P-value | % of Targets | % of Background | Motif file |
| 1 | 0.796 | A G T C A G C T A G C T A G T C A G C T A G C T A G T C A G C T A G C T A G T C | 1e-603 | -1389.699110 | 28.60% | 4.10% | motif file (matrix) |
| 2 | 0.766 | A C G T G A C T G A C T G C A T A T G C G A T C G C A T T A G C A G T C C G A T G A T C G A C T | 1e-181 | -418.570196 | 18.09% | 5.54% | motif file (matrix) |
| 3 | 0.883 | T A G C A G T C A C G T C G A T A G T C A T G C G T A C A G T C A G C T A G C T A G T C | 1e-70 | -162.322949 | 6.03% | 1.56% | motif file (matrix) |
| 4 | 0.813 | C T A G C T A G C T G A C T A G C T A G C T A G C T G A C T G A C T A G C T A G | 1e-37 | -86.468005 | 21.88% | 14.64% | motif file (matrix) |
